# Supplementary material for: Highly stretchable and transparent ionic conducting elastomers
Source: Nat Commun. 2018 Jul 6;9:2630. doi: 10.1038/s41467-018-05165-w (PMC6035269; doi:10.1038/s41467-018-05165-w)
Supplement: Supplementary file 1 — Supplementary Information [file 41467_2018_5165_MOESM1_ESM.pdf]

Supplementary Information

Ionic conducting elastomers

Lei Shi, Tianxiang Zhu, Guoxin Gao, Xinyu Zhang, Wei Wei, Wenfeng Liu, Shujiang Ding*

Department of Applied Chemistry, School of Science, MOE Key Laboratory for Nonequilibrium Synthesis and Modulation of Condensed Matter, State key laboratory of electrical insulation and power equipment, Xi'an Jiaotong University, Xi'an 710049, People's Republic of China

E-mail: dingsj@mail.xjtu.edu.cn

Supplementary Figures

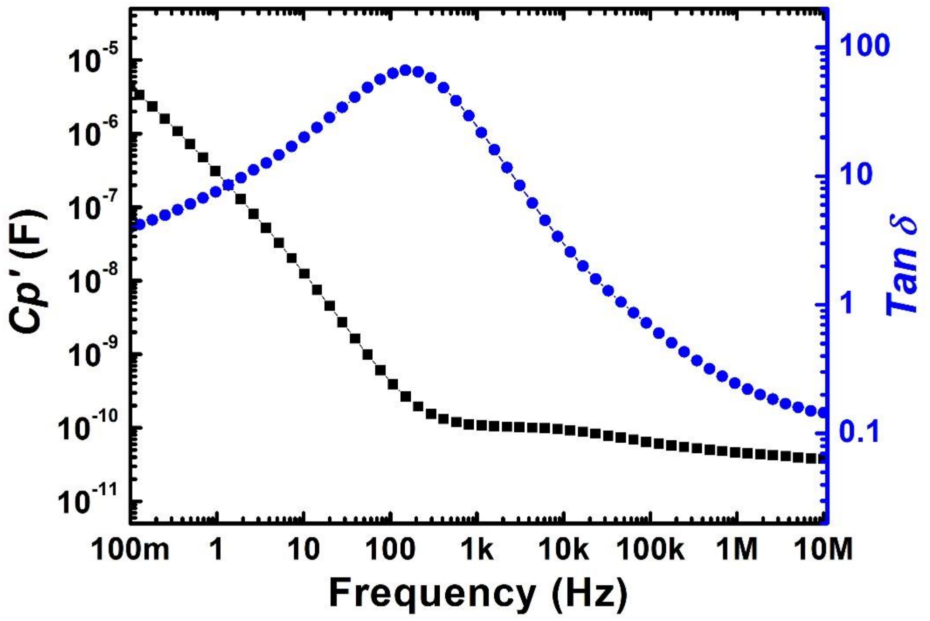

Supplementary Fig.1. Plot of capacitance (*Cp'*) and dielectric loss (*Tan δ*) versus testing frequency of ICE.

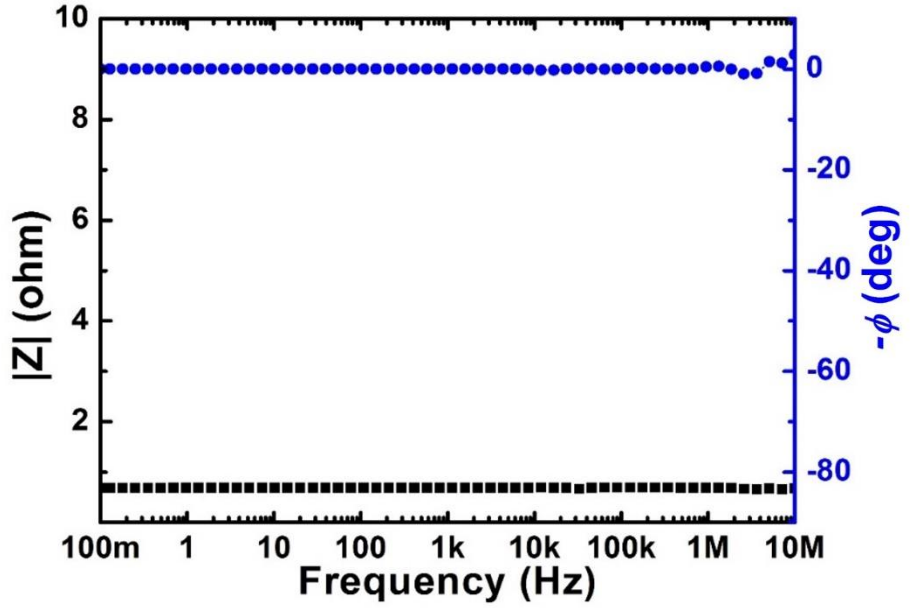

Supplementary Fig.2. Plot of impedance magnitude ($|Z|$) and negative phase angle ($-\phi$) versus testing frequency of carbon sponge.

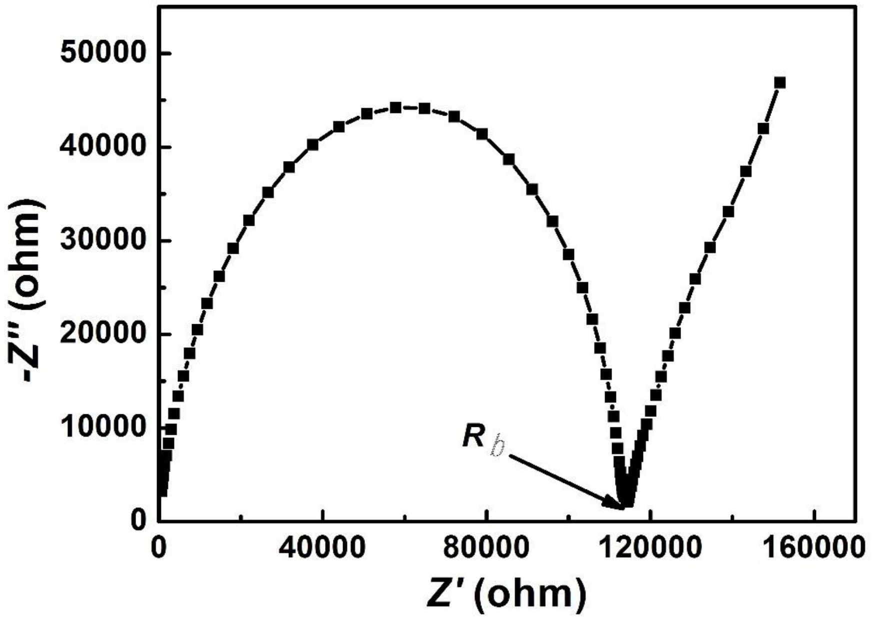

Supplementary Fig.3. Nyquist plot of impedance spectrum of ICE at room temperature. R_b represented the bulk resistance of testing ICE.

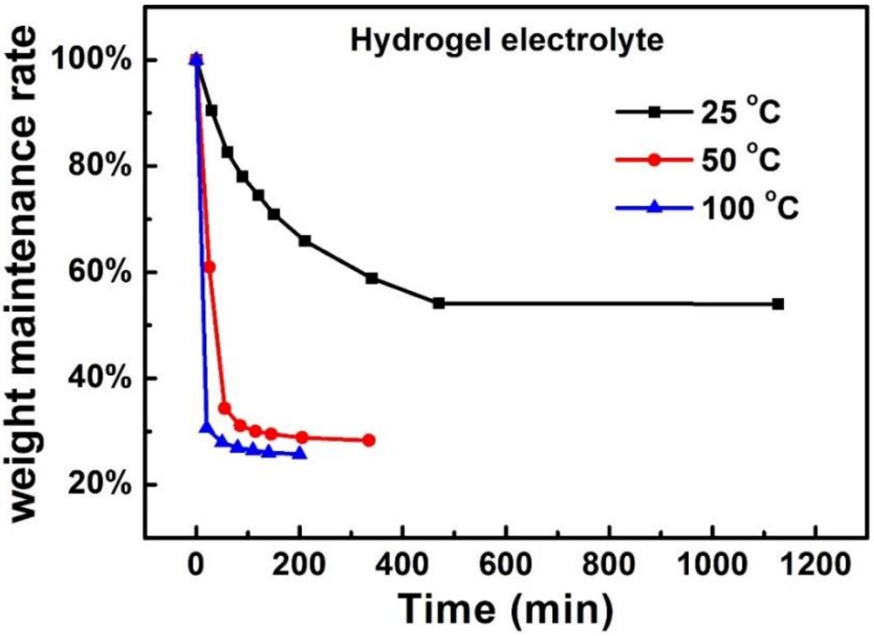

Supplementary Fig.4. Environmental stability testing of hydrogel electrolyte at open air. Plots of weight maintenances of hydrogel electrolyte at different temperature versus time.

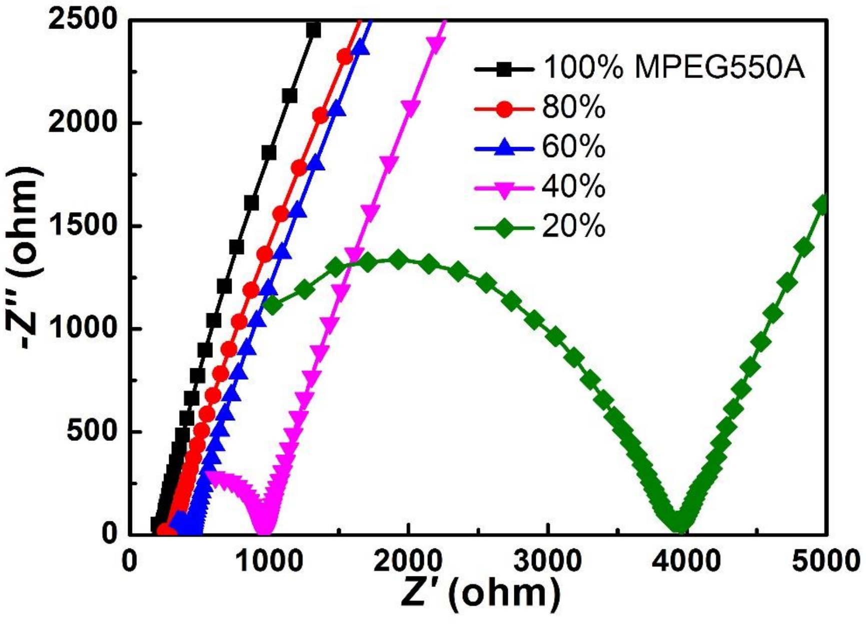

Supplementary Fig.5. Nyquist plots of impedance spectrum of copolymer ICEs at room temperature. The percentages represent volumes of MPEG550A to the total monomer volumes.

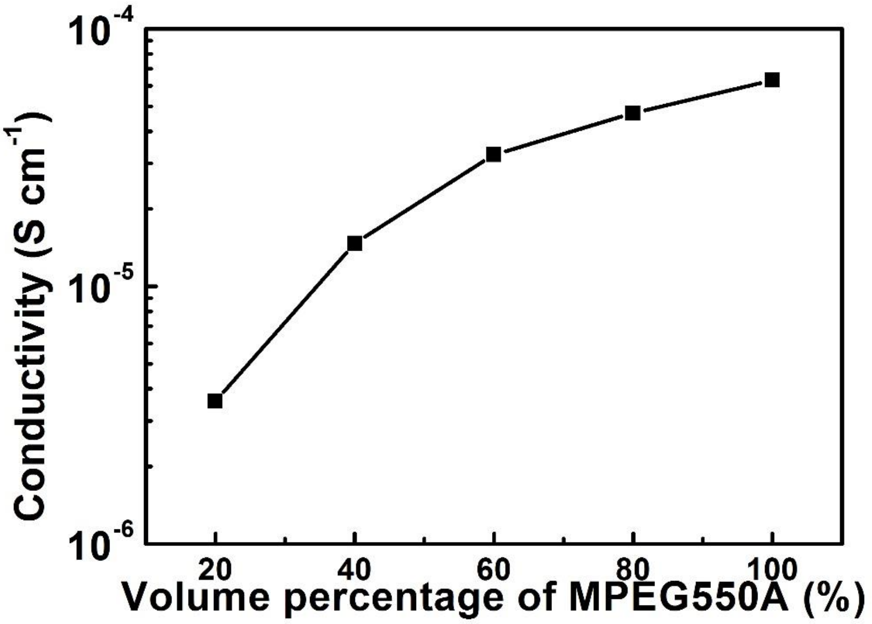

Supplementary Fig. 6. Conductivities of the copolymer ICEs with different MPEG550A volume percentages.

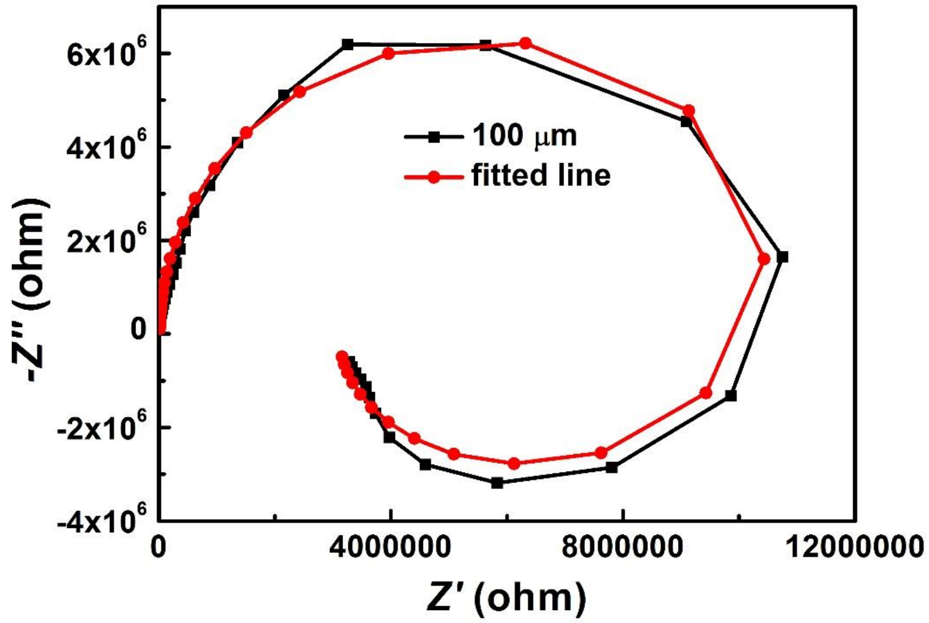

Supplementary Fig.7. Nyquist plots of impedance spectra of 100 μm thickness film covered touch sensor. The red line was the line of fitted results. The frequency range was 1 MHz-1 KHz. The insert was the fitted circuit.
