# Supplementary material for: Highly stretchable and transparent ionic conducting elastomers
Source: Nat Commun. 2018 Jul 6;9:2630. doi: 10.1038/s41467-018-05165-w (PMC6035269; doi:10.1038/s41467-018-05165-w)
Supplement: Supplementary file 3 — Description of Additional Supplementary Files [file 41467_2018_5165_MOESM3_ESM.pdf]

Description of Additional Supplementary Files

File Name: Supplementary Movie 1
Description: Demonstration of the adhesive property of ICE, when two pieces of ICE is attached together, the formed junction is hard to stretch apart.

File Name: Supplementary Movie 2
Description: A sensor detecting deformation by connecting to a LCR meter.

File Name: Supplementary Movie 3
Description: Demonstration of the sensitivity of the covered touch sensor
